# Exploring the link between Turkish gifted children’s perceptions of the gifted label and emotional intelligence competencies

**DOI:** 10.1038/s41598-022-17966-7

**Published:** 2022-08-12

**Authors:** Hülya Tercan, Müdriye Yıldız Bıçakçı

**Affiliations:** 1 Faculty of Health Sciences, Child Development Department, Hacettepe University, Ankara, Turkey; 2 Faculty of Health Sciences, Child Development Department, Ankara University, Ankara, Turkey

**Keywords:** Psychology, Environmental social sciences

## Abstract

The present study attempts to explore the relation between Turkish gifted children’s perceptions of the gifted label and their emotional intelligence competencies. We included 122 gifted children in this correlational study in the 2018–2019 academic year and collected the data using the Perceptions of Gifted Label Scale (PGLS) and the Emotional Intelligence Competencies Scale (EICS). In the analysis, we utilized descriptive statistics and calculated Pearson’s correlation coefficients between the variables. The mean age of the children was 11.5 years, and there was an equal number of girls and boys. The findings revealed that the children got almost average scores on all subscales of the PGLS. The results uncovered that self-perception of the gifted label was significantly correlated with friends’ and parents’ perceptions of the gifted label [*r* = 0.380, *p* < .01]. We found a significant negative relationship between the PGLS self-perception and the EICS self-consciousness. To put it more clearly, as having increased self-consciousness, they are likely to have decreased perception of being labeled decreases. . Our findings also seem noteworthy in suggesting a helpful conceptual framework for designing therapeutic interventions for gifted children, who are often considered more sensitive to social-emotional issues.

## Introduction

Definitions of giftedness are fundamentally built on two central tenets through a limited framework^1,2^: a natural potential ongoing since childhood and superior performance in skills that require cognitive abilities^3,4^. However, broader approaches to giftedness^5^ do not limit it to innate genetic influences. According to Heller and Perleth^6^, giftedness should not be considered in terms of only its cognitive aspects. Giftedness, as a whole, includes creativity, talent, diverse personality traits, and social-emotional states^7,8^. Although scholars from different cultures seem to have brought different definitions for giftedness, they all highlighted changing intelligence perceptions over time to describe gifted individuals^9^. Later on, as the intelligence quotient (IQ) test was largely associated with giftedness^10^ an IQ score of 120 and above on the Wechsler or Stanford-Binet Intelligence scale became a significant starting point for identifying gifted children^11,12^.Recent years have witnessed substantial research interest in emotional intelligence and more comprehensive studies on giftedness and talent. The novel understanding of giftedness is now based on many psychologists’ extending the traditional definition of giftedness to include not only cognitive abilities but also the regulation of experience, self-expression, and emotions^13–15^.

The previous research documented that gifted children think and behave differently from their peers, which may even be observed at the initial stages of their development^8,16,17^. Gifted children are generally thought to have robust features regarding perceptual reasoning, auditory perception, and visuospatial thinking^10^. Nevertheless, findings on the gifted's developmental characteristics may vary in the literature. While some studies that appreciate the results of IQ tests showed that gifted children perform better in working memory (WM) and speed of processing (SP) than typically developing children, there are also findings showing that these children may have difficulties in SP^18^. Based on these findings, it may be claimed that gifted children receive more stimuli and perceive their environment better thanks to their enhanced sensory awareness, which is associated that they may adopt a certain attitude toward acquiring knowledge and understanding the world^19,20^.

Nevertheless, such unique characteristics of gifted children can make them more vulnerable. Unlike their peers, they may have anxiety and emotional and personality issues, which may hinder them from exhibiting their superior potential^21–25^. In this respect, there may be a dichotomy between the psychological resilience of gifted children and their emotional fragility^26,27^.Supporting this view, some scholars and psychologists emphasized that gifted children are at risk of developing emotional difficulties in internal, environmental, and interpersonal interactions, which may adversely affect their peer and sibling relationships^28–30^. On the contrary, there is a lasting idea that the gifted have higher-order cognitive and psychosocial abilities that can take them to achievement^31^.

In a recent review, Papadopoulos^27^ emphasized that instant access to information and increased conscious awareness of environmental stimuli in today's world can lead gifted children to deal with the problems of humanity from an early age. Yet, concentrating on such issues may elevate stress and emotional sensitivity among the gifted^32^. Therefore, despite the fact that some of their enhanced abilities can help in problem-solving, being gifted may bring some psychological problems by increasing the risk of social isolation and stigma^33^ While some studies reported that gifted adolescents are particularly prone to anxiety and mood disorders^34,35^ recent studies showed that gifted children can be as psychosocially compatible as their classmates when confronting with socio-emotional problems^36,37^. Thus, the research interest has been shifted to explore the emotional characteristics of gifted children in the last two decades^38^. The unprecedented interest in research on gifted children may largely be attributed to the idea that the emotional and social characteristics of the gifted have a greater impact than expected on both their talent development and general well-being^39^ which may be linked with their emotional intelligence.

Emotional intelligence (EI) refers to a set of core competencies and skills organized on recognizing, expressing, processing, and regulating one’s own emotions and those of others. Jack Mayer and Peter Saloyev^12^ carried out the first systematic study on EI in the late 1990s. Then, the concept gained increasing popularity in the following years thanks “Emotional Intelligence,” the book published by Goleman^40^. Since then, EI research has been especially appreciated for the benefit of gifted children who are often considered more vulnerable to social-emotional issues^41^. Studies on the subject revealed that EI supports the social-emotional development of gifted school-age children and, thus, contributes to their resilience and adaptation^38,42–44^. These studies explored the impacts of emotional intelligence and social-emotional development on the relationship between giftedness and resilience and emphasized some shared individual characteristics between resilience and giftedness. Moreover, they discussed that emotional intelligence is an influencing factor on resilience and pointed out that some traits utilized to describe individuals with social-emotional resilience can also be used to describe the gifted. Examples of such common traits include success in assuming a task, reflectiveness, a desire to learn, maturity, an internal locus of control, risk-taking, and self-understanding. In addition, children with high EI may have more positive relationships, are less likely to take risks, experience fewer emotional ups and downs, and show better academic performance^45–47^. Therefore, the research on EI may provide a better understanding of the associations between the gifted’ characteristics of emotional functioning and their cognitive and social-emotional competencies^48,49^. For this reason, we thought that it would be heplful to consider emotional intelligence with the impact of labeling.

Labeling, on the other hand, may be conceived of as classifying children by several indicators (e.g., intelligence quotient, standard test scores, or test achievement). In making such a classification, experts often hope to be able to explain children’s different needs or strengths. In fact, labeling is a tendency to influence adults’ perceptions of the child and the child’s self-perception. Hence, regarding the social consequences of the gifted label, it is deemed necessary to uncover the perceptions of those with whom the gifted interact, as well as the perceptions of those with the gifted label. Labeling theory^50^suggests that the labeled has a symbiotic relationship with their environment. The initial research of Rosenthal and Jacobson^51^ based on the Pygmalion effect highlights that students’ performance is likely to be affected by teachers’ perceptions of those students. Teacher perceptions usually manifest in their interactions with students in the classroom environment. For example, if a teacher perceives that any child in the class is capable of exhibiting superior academic performance, they are more likely to expect high achievement from that child. The theory is largely confirmed in subsequent research^52–54^. Indeed, such discussions on the gifted label aim to show that giftedness is not deviance but the power shining out on one’s behavior and socialization. Yet, it should be noted that high expectations with the gifted label may result in social isolation, adverse emotional consequences, and a disparity between expectations and performance. In this respect, the gifted label has the potential to affect a child’s life through external choices, behavior, and opportunities offered^55^.

Ultimately, the present paper attempts to explore two issues closely intertwined with the above-mentioned situations. Accordingly, we investigated the association between EI competencies and some positive and negative outcomes of gifted children’s perceptions of the gifted label. We hope the results lead professionals working in the field of giftedness to make their decisions considering only gifted children’s development and EI characteristics.

## Method

The present research employed a correlational design to explore the association between Turkish gifted children's perceptions of the gifted label and their emotional intelligence competencies.

### Study Setting and participants

We only included voluntary children with a diagnosis of giftedness aged 10–13, attending a center for gifted education, without any developmental or medical diagnosis other than the diagnosis of giftedness, and with at least one sibling. We carried out this study in three centers for gifted education (Science and Art Center—BILSEM) affiliated with the District Directorate of National Education in Ankara in the fall semester of the 2018–2019 academic year. BILSEMs in Turkey are affiliated with the Ministry of Education and serve to support the needs of the gifted in Turkey. In the Turkish education system, BILSEMs administer traditional intelligence tests and admit students with an IQ score of 130 and above.

First, we contacted the administrators and teachers of these centers and provided them with brief information about our study. Then, we held an informatory meeting to explain the objective and content of the study to children and their parents. Those who satisfied our inclusion criteria and accepted to participate in the study voluntarily were asked to fill out an informed consent form. The sample included 122 voluntary students attending three BILSEMs. We calculated the sample size using g-power analysis and did not request any participation fee from the participants.

The mean age of the participating children was 11.5 years, and half of them were females.. Most of the children’s mothers (56.6%) had a primary school degree, while the majority of their fathers (54.9%) held an undergraduate degree. More than half of the students (61.5%) had at least one sibling. Yet, children without siblings were not included in the study (Table 1).

**Table 1:** Participants descriptive characteristics.

Descriptive characteristics		n	%
Gender	Female	61	50.0
	Male	61	50.0
Age	10 years	26	21.3
	11 years	33	27.0
	12 years	33	27.0
	13 years	30	24.6
Maternal education	Primary-high school degree	69	56.6
	Undergraduate-postgraduate degree	53	43.4
Paternal education	Primary-high school degree	55	45.1
	Undergraduate-postgraduate degree	67	54.9
Number of siblings	1	75	61.5
	2	46	37.7
	3 or more	1	0.8
	Total	122	100

### Measures

We collected the data using a questionnaire booklet that covers a sociodemographic information form, the Perceptions of Gifted Label Scale (PGLS), and the Emotional Intelligence Competencies Scale (EICS). The data collection process was based on the children’s self-reports and was performed in a classroom environment under the supervision of the researchers.

#### Perceptions of gifted label scale (PGLS)

We previously developed the PGLS to provide a multidimensional evaluation of gifted children’s perceptions of being labeled after the diagnosis.

The PGLS consists of thirty-six items rated on a 4-point Likert-type scale ranging from “Never” (1) to “Always” (4). It includes five subscales designed to reveal how the gifted label is perceived by the child, friends, parents, teachers, and siblings. Instead of the total score, subscale scores are considered for measurements. Thus, a high score on a subscale indicates a stronger perception of the gifted label on that dimension, while a low score refers to a poorer perception of the gifted label.

We performed its validity and reliability study with children aged 10–13. First, we evaluated its content validity by referring to expert opinions based on the Lawshe technique and calculated its content validity ratio (CVR) to be 0.82. Then, we explored its construct validity and performed confirmatory factor analysis (CFA). While developing the scale, we created a five-factor model relying on relevant theories and expert opinions and tested the model with CFA on the LISREL (ver.8.80) software. Accordingly, it was determined that the difference between the observed and expected asymptotic covariance matrices (χ2 value) was significant at 0.01 (Satorra-Bentler Scaled χ2 = 1210.86 *p* = 0.00). The root mean squared error of approximation (RMSEA) value became 0.067. An RMSEA value lower than 0.08 indicates good fit^56^. Moreover, we considered Comparative Fit Index (CFI) and Norm-Free Fit (NNFI) to decide on the model-data fit. Overall, the high values of the mentioned fit indices (0.89 and 0.88, respectively) suggested that the model fit well with the data.

For reliability concerns, we calculated Cronbach’s alpha coefficients and found the coefficients to be above 0.70 for each subscale, indicating the scale reliably measure the intended construct.

#### Emotional intelligence competencies scale (EICS):

Titrek^57^ developed the scale to measure the emotional competencies of school-age children. The scale consists of 72 items within five subscales: self-consciousness, emotion manipulation, emotion motivation, empathy, and social skills. The EICS is scored on a five-point Likert-type scale ranging between “Never” (1) and “Always” (5). The reliability coefficients for the scale were calculated to be 0.80 for the self-consciousness, 0.73 for the emotion manipulation, 0.76 for the emotion motivation, 0.88 for the empathy, 0.88 for the social skills, and 0.96 for the total score.

### Statistical analysis

We analyzed the data using descriptive statistics (numbers, percentages, means, and standard deviations) and a bivariate correlation test on the SPSS 22.0 program. We checked the Kolmogorov–Smirnov values to see if the data showed a normal distribution. To explore relationships between the participants’ scores, we calculated Pearson’s correlation coefficients for normally distributed data and Spearman’s correlation coefficients for non-normally distributed data. Moreover, to understand if the scores differed by some variables, we performed independent samples t-test and one-way analysis of variance (ANOVA) (Mann–Whitney U and Kruskal–Wallis tests for non-normally distributed data). A *p*-value < 0.05 was accepted as statistically significant. Finally, we interpreted the correlation coefficients as weak relationship (0.00–0.25), moderate relationship (0.25–0.50), moderate-good relationship (0.50–0.75), and strong relationship (over 0.75)^58^.

### Ethical considerations

The Research Ethics Committee of Ankara University granted the relevant approval to our study (Number: 85434274-05.04.04, Decision number: 25/323, Date: November 28, 2016). We carried out the study in October–November, 2018. This research was performed in accordance with the Declaration of Helsinki, and informed consent was obtained from all participants and/or their legal guardians.

## Results

Table 2 presents the participants’ mean scores on the PGLS subscales. Accordingly, the children scored close to averages on the PGLS parents (8 items; *M* = 16.713), teachers (7 items; *M* = 15.418), and siblings (6 items; *M* = 14.237). Yet, they got above-average scores on the PGLS self-perception (7 items; *M* = 15.475) and friends (8 items; *M* = 15.467). When it comes to the EICS, we found that the children scored close to the averages on the EICS self-consciousness (12 items; *M* = 38.860), emotion manipulation (15 items; *M* = 54.000), emotion motivation (14 items; *M* = 45.106), empathy (12 items; *M* = 38.319), and social skills (16 items; *M* = 68.655). The mean scores obtained on EICS social skills, emotion manipulation, and emotion motivation were higher than on the other subscales. In addition, the lowest mean scores were on the EICS empathy and self-consciousness. Nevertheless, the children’s EICS mean total score was above the average (72 items; *M* = 244.942).

**Table 2:** Descriptives for the perceptions of the gifted label (PGLS) and emotional intelligence (EICS).

Variable	Mean	SD
**PGLS**		
Self-perception	15.475	3.064
Friends’ perceptions (Friends)	19.467	2.227
Parents’ perceptions (Parents)	16.713	2.938
Teachers’ perceptions (Teachers)	15.418	2.237
Siblings’ perceptions (Siblings)	14.237	2.093
**EICS**		
Self-consciousness	38.860	4.676
Emotion manipulation	54.000	4.704
Emotion motivation	45.106	4.410
Empathy	38.319	3.321
Social skills	68.655	5.071
Total score	244.94	17.272

Since the main variables of the study were found to be normally distributed, we calculated *Pearson’s correlation coefficients* to reveal the association between them. As in Table 3, we found a *significant positive relationship* between the children’s scores on the PGLS self-perception and friends [*r* = 0.380, *p* < 0.01]. Therefore, gifted children’s stronger perception of the gifted label may apply to their friends’ perceptions. In addition, there was a *significant positive correlation* between the PGLS self-perception and parents [*r* = 0.292, *p* < 0.01]. However, we discovered some differences between teachers’ and parents’ perceptions of the gifted label; the PGLS teachers was *negatively correlated* with the PGLS parents [*r* = −0.422, *p* < 0.01]. In other words, while the parents have an increased perception of the gifted label, it is likely to be vice versa among teachers. Considering the findings in Table 4, we concluded significant positive relationships between all the EICS subscales.

**Table 3:** Correlations between the PGLS subscales.

PGLS Subscales	Self-perception	Friends	Parents	Teachers	Siblings
Self-perception	–	0.380**	0.292**	0.265**	0.249**
Friends		–	0.334**	0.330**	0.215*
Parents			–	0.-422**	0.104
Teachers				–	0.065
Siblings					–

*p < 0.05.

**p < 0.01.

**Table 4:** Correlations between the EICS subscales.

EICS Subscales	Self-consciousness	Emotion manipulation	Emotion motivation	Empathy	Social skills
Self-consciousness	–	0.533**	0.557**	0.318**	0.480**
Emotion manipulation		–	0.599**	0.479**	0.553**
Emotion motivation			–	0.411**	0.553**
Empathy				–	0.553**

*p < 0.05.

**p < 0.01.

Table 5 demonstrates the correlations between the EICS and the PGLS subscales. Accordingly, we found a *significant negative* association between the EICS self-consciousness and the PGLS self-perception [*r* = −0.238, *p* < 0.01], which may indicate that the more self-consciousness the gifted children develop, a stronger perception of the gifted label they have.

**Table 5:** Correlations between the PGLS subscales and EICS subscales (n = 122).

EICS/PGLS Subscales	Self-perception	Friends	Parents	Teachers	Siblings
Self-consciousness	−.238**	−0.010	−0.120	0.040	−0.162
Emotion manipulation	−0.164	−0.120	−0.123	−0.098	−0.160
Emotion motivation	−0.152	−0.144	−0.130	−0.138	0.046
Empathy	0.052	0.143	0.030	0.022	−0.018
Social skills	0.037	−0.070	−0.070	−0.023	−0.045

*p < 0.05.

**p < 0.01.

Table 6 shows the comparisons of the scores on both scales by age and gender. Accordingly, the scores on the PGLS self-perception [*t*(120) = 0.353, *p* > 0.05], parents [*U* = 1809.500; *p* > 0.05], teachers [*U* = 1773.500; *p* > 0.05], friends [*t*(111.4) = 1.263, *p* > 0.05], and siblings [*t* (0.734), *p* > 0.05] did not significantly differ by gender. Yet, the children *significantly differed* on the PGLS self-perception by age [χ^2^ (*df* = 3, n = 122)]. Considering the mean ranks of the age groups, the children aged 10 years got the highest scores on the PGLS self-perception. Similarly, there were *significant differences* between the children’s scores on the PGLS friends by age [F(3.118) = 3.018; *p* < 0.05]. In this study, the children did not significantly differ on the PGLS parents [χ^2^ (*df* = 3, n = 122)], teachers [χ^2^ (*df* = 3, n = 122)], and siblings [F(2.588); *p* > 0.05].

**Table 6:** The comparisons of the scores on PGLS and EICS subcategories by age and gender.

		PGLS subcategories					EICS subcategories					
Age		Own Per.	Friends	Parent	Edu.	Sibling	Self con.	Emo. Manip.	Emo. Motiva	Empathy	Social skills	Total Score
	P	0.017	.033	0.839	0.949	.056	0.000	0.002	.074	0.024	0.087	0.000
10 ${\overline{\text{X}}}$		15.5942	20.3846	16.9565	15.3913	20.3846	38.7391	53.4203	43.1923	44.8696	68.2319	243.5797
11 ${\overline{\text{X}}}$		14.7879	19.1212	16.6667	15.4545	19.1212	39.3636	54.1212	45.3636	38.0909	68.0303	244.9697
12 ${\overline{\text{X}}}$		14.8788	19.7273	16.8182	15.2727	19.7273	39.4848	54.7576	45.3939	38.6364	68.9697	247.2424
13 ${\overline{\text{X}}}$		15.4000	18.7667	16.3000	15.4000	18.7667	40.6000	55.8333	46.1667	39.4000	70.2667	252.2667

Gender												
	p	0.725	0.209	.792	.652	0.464	0.072	.000	0.183	0.011	0.004	0,002
	t/U	**t=**0.353	**t=**1.263	**U=**1809.500	**U=**1773.500	**t=**0.734	**t=** 1.817	**U=** 1126.500	**t=**1.339	**t=**2.595	**U=**1302.500	**t=**3.097
Female ${\overline{\text{X}}}$		15,377	19.213	60.66	62.93	14.098	39.623	73.53	45.639	39.082	70.65	249.623
Male ${\overline{\text{X}}}$		15,573	19.721	62.34	60.07	14.377	38.098	49.47	44.574	37.557	52.35	240.262

*p < 0.05.

**p < 0.01.

The findings revealed that the scores on the EICS self-consciousness [*t*(1.817); *p* > 0.05] and emotion motivation [*t*(1.339); *p* > 0.05] did not differ significantly by gender. On the other hand, the EICS empathy [*t*(1.339); *p* < 0.05), emotion manipulation [*U* = 1126.500; *p* < 0.05], social skills [*U* = 1302.500; *p* < 0.05], and the EICS total score [*t*(3.097); *p* < 0.05] significantly differed by gender. Accordingly, the girls got higher scores on these subscales than boys (Table 6).

The EICS self-consciousness [*F*(3.118) = 7.403; *p* < 0.05], emotion manipulation [χ2 (*df* = 3, n = 122)], empathy (χ2 (*df* = 3, n = 122)], and the EICS total score [χ2 (df = 3, n = 122)] *significantly differed* by age [*F*(3.118) = 6.567; *p* < 0.05]. Considering the mean ranks of age groups, the children aged 13 years had the highest scores on the subscales. Moreover, we performed a Schefee test to reveal the source of differences and found that the differences between the age groups of 10–11 years, 10–12 years, and 10–13 were significant (*p* < 0.05). However, the children did not significantly differ on the EICS social skills and emotion motivation [χ2 (*df* = 3, n = 122)], [*F*(2.373) = ; *p* > 05].

## Discussion

The present study attempts to explore the relation between Turkish gifted children’s perceptions of the gifted label and EI competencies.

The findings on the perception of the gifted label revealedthat the gifted children, their friends, and their parents have positive perceptions of the gifted label and that such positive perceptions have a bidirectional relationship. The parental emphasis of “giftedness” on a gifted child may lead the child to have and reflect a stronger perception of being gifted. Therefore, it is not surprising that the increase in the child’s perception of the gifted label promotes such a perception among their friends and siblings. The literature on friendship relations documented that gifted children are likely to experience social isolation and feel less appreciated by their friends, even if their peers accept them as popular^60–62^. In addition, it was reported that gifted children tend to make older friends with whom they feel mentally close rather than spending time with their chronological peers^63^ and experience less intimacy in their friendships^64^. A study by Coleman and Cross^65^ explored the social acceptance of gifted adolescents in their high school years and conducted interviews with the young attending a special summer program. Accordingly, the participants showed being gifted as a barrier to full social acceptance and as a social handicap. Moreover, some participants reported that they used to conceal their giftedness and be less visible.

In this study, it is noteworthy that there was a positive correlation between children’s self-perception and their parents’ perceptions of the gifted label. According to Gates^55^ although labels help educators to adequately describe specific characteristics of a child (e.g., giftedness, specific learning disability, or attention deficit hyperactivity disorder) and meet their individual needs, the overemphasis on labels may devalue the child over time^66^. The fact that labeling is more common in centrally funded education systems (e.g., the USA) is thought to be related to the distribution of federal funds to public schools where only certain diagnostic groups are categorically defined^67^.

Another remarkable finding of the present research is that the parents’ perceptions of the gifted label were negatively correlated with the teachers’ perceptions, which may be because the parents’ perceptions of the giftedness of their children may not seem realistic to the teachers^68^. Blass^69^ pointed out that gifted children are often categorized in a diverse minority group with high abilities whose needs are unrecognized and unmet. He also emphasized that these children may experience a range of social-emotional difficulties (e.g., peer exclusion, isolation, stress, anxiety, depression, and destructive perfectionism). The literature also indicates that some gifted children may experience failures in school and that many teachers have difficulty recognizing and meeting their needs^70^.

In this study, we also explored gifted children’s EI and found that the participating children scored close to the average on the EICS subscales, except for the self-consciousness and empathy, and got quite high total scores. Therefore, it would be helpful to support gifted children with empathy and self-consciousness, which are considered vital components of EI. The literature hosts several studies having traced EI between groups with and without a gifted diagnosis. Although many studies revealed a positive link between gifted children’s social-emotional development and EI and many difficulties experienced^63,71,72^ we still have limited knowledge of how these variables affect each other.

Our results showed that the perception of the gifted label did not significantly differ by gender. On the other hand, the perception of the gifted label significantly differed by age.. Accordingly, we found that the 10-year-olds had the highest perception of the gifted label, which overlaps the previous findings suggesting that one’s feelings and thoughts about giftedness change with age Undoubtedly, there may be many reasons for these results, including developmental characteristics. Children in this age group begin to develop more awareness of their own characteristics and environment. In addition, being more self-directed with the onset of adolescence may lead them to adopt a greater perception of giftedness compared to older age groups and to demonstrate more giftedness toward their family and peers.

However, it is emphasized that the fresh diagnosis and the emphasis by the family and environment on the diagnosis may reinforce the perception of the gifted label among younger children.

On the other hand, the gifted children’s EICS total and subscale scores (emotion manipulation, empathy, and social skills) significantly differed by gender. We found that the girls got significantly higher EICS total and subscale scores than boys. In addition to gender, the participants’ EICS total and subscale scores (except for emotion motivation) significantly differed by age. Accordingly, while the 13-year-olds had the highest total and subscale scores, the 10-year-olds got the lowest mean scores. In parallel with our findings, the previous research showed that EI is highly correlated with age; the probability of having higher EI increases with age^73^.This situation is thought to be because EI is a developing ability, and accumulated life experiences may contribute to the development of EI.

Regarding the correlations between the PGLS and EICS scores, we found a significant negative association between the children’s self-consciousness and self-perception of the gifted label. In other words, the increased self-consciousness among children may hinder their self-perception of the gifted label. In the literature, self-consciousness is considered a ground on which all tenets of EI are built. A remarkable meta-analysis study examined 1,234 experimental studies on giftedness that were published between 1998 and 2010^74^. While 334 of these studies were related to educational practices and 194 were on creativity, only 15 were conducted on counseling gifted children. However, almost no studies attempted to unveil the association between the gifted label and EI among gifted children. While there is a limited number of studies on emotional development about how the gifted label is perceived by gifted children, their parents, and others, there are no studies exploring the direct association between EI and the gifted label. Therefore, our results would make a substantial contribution to the literature.

## Conclusion

Overall, our findings suggested that although the gifted label has positive aspects, it may bring some psychological, emotional, and social obligations. Moreover, the findings in this study need to be minded to grab an in-depth understanding of gifted children's development and to realize that these children's strong emotional reactions and sensitivities may be a critical aspect of their personality development. The strengths of the present research may be that it simultaneously evaluated the association between emotional intelligence and labeling in the context of gifted children's social-emotional development and explored the multidimensional impact of labeling. In this respect, we believe that our findings may guide experts and families in determining the most appropriate developmental approaches to gifted children.

On the other hand, the current study's main limitation is that it is cross-sectional. Therefore, it is not appropriate to make a causal inference. One may obtain diverse findings if carrying out the research—in the same research design—in a different time period or with a different sample group. Additionally may be the marked differences between countries and cultural contexts regarding definitions of "gifted" and diagnostic criteria. There is still no consensus in the literature on acceptable criteria for defining children as gifted or high achievers and how well these criteria describe the same construct. We carried out this study only with a group falling within the definition of gifted in Turkey. Further research may reveal the relevance of labeling and emotional intelligence in other cultures. In addition, longitudinal studies would contribute more to the field of giftedness to make accurate assessments and develop relevant policies for practice and education.

## Data Availability

The datasets used and/or analyzed during the current study are available from the corresponding author on reasonable request.
